# Association of brachycephaly, sleep-disordered breathing, and inflammatory response in dogs

**DOI:** 10.1093/jvimsj/aalag126

**Published:** 2026-07-20

**Authors:** Iida Niinikoski, Minna M Rajamäki

**Affiliations:** Department of Equine and Small Animal Medicine, University of Helsinki, Helsinki, Finland; Department of Equine and Small Animal Medicine, University of Helsinki, Helsinki, Finland

**Keywords:** apnea–hypopnea index, brachycephalic obstructive airway syndrome, low-grade inflammation, obstructive sleep apnea, obstructive respiratory event index

## Abstract

Sleep is fundamental to the welfare of all animals, including dogs. Various important processes take place during sleep, and while complete sleeplessness leads to death, sleep fragmentation is also harmful. Brachycephaly, the severely shortened snout and skull in the absence of a concurrent decrease in the volume of the soft tissues of the upper airways, causes many welfare concerns, including sleep-disordered breathing (SDB). In obstructive SDB, the most common form of SDB in dogs, repeated episodes of partial or complete blockages of airflow occur during sleep. This leads to intermittent hypoxemia. Sleep-disordered breathing resembles obstructive sleep apnea in humans, which is associated with various comorbidities and a higher mortality rate. Intermittent hypoxemia is associated with chronic low-grade inflammation, an independent risk factor for a higher mortality rate in people. Research into SDB in dogs has been limited, largely due to arduous diagnostic methods, but in recent years, new developments have taken place. Risk factors for SDB include brachycephaly, moderate or severe signs of brachycephalic obstructive airway syndrome, and excess weight. As SDB negatively affects welfare on multiple levels and is at least partly treatable with surgical and conservative methods, easier methods are needed to diagnose the individuals in need of treatment. On a population level, the future of brachycephalic breeds needs to be critically evaluated.

## Introduction

Sleep is an integral part of welfare and wellbeing. In recent years, research into sleep-disordered breathing (SDB) in dogs has increased awareness of the condition, prevalent in brachycephalic dogs. In brachycephaly, the lack of a decrease in the volume of upper airway soft tissue ^1^^,^^2^ despite a reduction in skeletal muzzle length^3^ results in a varying degree of upper airway obstruction during both wake and sleep states. In obstructive SDB, which resembles obstructive sleep apnea (OSA) in humans, episodes of hypopnea (shallowed breathing) and apnea (complete cessation of airflow) occur due to upper airway obstruction.^4^^,^^5^ In humans, OSA is associated with many comorbidities, such as higher cardiovascular risk.^6^ In addition, when untreated, severe OSA in humans increases the mortality rate^7^ and leads to chronic low grade inflammation,^8^ which is an independent risk factor for a greater total mortality rate^9^ and various chronic disorders.^10^

Sleep-disordered breathing in dogs was first described in brachycephalic English Bulldogs in 1987 using polysomnography,^4^ the gold standard for SDB diagnostics in humans.^11^ In addition, whole-body barometric plethysmography^12^ and a portable neckband system^13^^,^^14^ are used for diagnosing SDB. Although there is some evidence of a proinflammatory condition associated with brachycephaly,^15–17^ low-grade inflammation has not been evaluated in connection with SDB.

## Sleep

Sleep is a necessity, as sleeplessness ultimately leads to death.^18^ Although the fundamental reasons behind sleeping remain unknown, many critical processes occur during sleep: replenishment of energy stores, biosynthesis and removal of toxic waste products, modulation of immune response and learning, and neurodevelopment and memory consolidation.^19^^,^^20^ Across species, sleep is strongly interconnected with wellbeing and welfare: the quality of sleep can be affected by a decline in welfare, and welfare can be negatively influenced by poor sleep quality.

### Regulation of sleep

Regulation between wake and sleep is controlled by specific neural circuits and nuclei in the brain, as different neurotransmitters are released in the different states of vigilance.^21^ In sleep, wake-promoting cells are silenced, and reciprocally, sleep-promoting cell groups are silenced during wake.^21^ The main processes controlling wake and sleep are circadian rhythm and homeostatic sleep pressure.^20^ The internally generated circadian rhythm regulates the timing of sleep, and the duration and intensity of sleep are controlled by homeostatic sleep pressure. Sleep-promoting circumstances in dogs include sleep debt,^22^ nighttime,^23^ and satiety.^24^ Wake-promoting circumstances include daytime^25^ and hunger.^24^

Domestic dogs are diurnal, that is, wake occurs mainly during the day and sleep primarily during nighttime.^23^^,^^24^^,^^26^ This diurnal activity pattern differs from non-domesticated canids^27^ and is thought to reflect adaptation to the sleep–wake pattern of humans during tens of thousands of years of domestication. Peak activity occurs around 7:00 and 19:00^23^ and total sleep time ranges from 8.4 to 13.5 h in adult dogs.^28–31^ Alongside sleeping during the night for 6-7 h,^32^^,^^33^ dogs also sleep during the afternoon for approximately 3 h.^32^ Daytime sleep is a normal, important part of the dog’s sleep–wake pattern.^32^

The duration and intensity of sleep are regulated by homeostatic sleep pressure, which is reflected in the length of sleep and neuronal electroencephalography (EEG) activity.^34^ During wakefulness, the need for sleep, also called sleep pressure, increases until it is high enough to impose sleep.^34^ Increasing sleep pressure can be identified as the accumulation of sleep-promoting substances, such as adenosine and nitric oxide, in cerebrospinal fluid.^34^ During sleep, sleep pressure dissipates until low enough for wake to commence. Lack of sleep increases total sleep time and the proportion of rapid eye movement (REM) sleep in dogs^22^ and affects EEG activity of different sleep stages.^25^ Sleep-deprived dogs are less alert and more inactive, eat more, and play less when awake compared to dogs with non-fragmented sleep.^33^

Age affects sleep–wake patterns, as older dogs sleep more than younger dogs^35^ and are less active during the day.^24^ Total sleep time is reduced and daytime sleep bouts more frequent in older dogs.^35^ Cognitive dysfunction in dogs, a disease process associated with aging, induces more pronounced changes to sleep–wake cycles, with higher time periods of restlessness and wandering during nighttime.^36^^,^^37^

### Sleep–wake cycle

Sleep is divided into 3 stages in dogs: drowsiness, non-rapid eye movement (NREM) sleep, and REM sleep.^25^ Each sleep stage is characterized by different waveforms in EEG.^25^ During drowsiness, the eyes of the dog can be open or closed and there is little motor activity,^25^ and in the absence of an EEG recording, drowsiness cannot be distinguished from wakefulness. Differences between the 4 states of vigilance (wake, drowsiness, NREM, and REM sleep) are presented in Table 1.

**Table 1:** Differences in observable and measurable parameters between the 4 vigilance states in dogs.

	**Wake**	**Drowsiness**	**NREM**	**REM**
***Movement***	Frequent^38^	Slow opening and closing of the eyes possible, dog may be sitting with eyes open^31^		Twitching of nose, lips, ears, whiskers, and limbs^39^^,^^40^
***Muscle tone***	Elevated^25^^,^^38^	Lowered but observable^38^	Decreased^38^^,^^41^	Muscular atonia^38^
***Cardiac function***	Highest heart rate in the different vigilance states^42^^,^^43^	Heartrate higher than in NREM and REM^43^	No difference in heartrate between sleep stages^42^	Irregular^25^^,^^38^
				Lowest heart rate^43^
		No difference in heartrate between sleep stages^42^		No difference in heartrate between sleep stages^44^
***Respiration***	Respiratory frequency depends on activity status^44^	Fairly regular^25^^,^^38^	Relatively regular^25^^,^^38^	Irregular^25^^,^^38^
		No difference in respiratory frequency between sleep stages^43^	No difference in respiratory frequency between sleep stages^43^	No difference in respiratory frequency between sleep stages^43^
***Electroencephalography***	Increased high frequency and fast activity^25^^,^^38^^,^^41^	Increased high frequency and fast activity^25^^,^^38^	≥15 μV δ waves: 1-4 Hz^25^^,^^38^ and/or sleep spindles: 12-16 Hz, duration ≥ 0.5 s^25^^,^^38^^,^^46^^,^^47^	Increased θ activity: 4.25-4.5 and 7-8 Hz^25^^,^^31^^,^^38^^,^^45^^,^^47^
	α waves: 8.75-12.75 Hz^38^	α waves: 8.75-12.25 Hz^25^^,^^38^^,^^45^		Fast activity,^25^^,^^31^^,^^38^^,^^45^ high frequency^22^^,^^41^
	β waves: 15-30 Hz^38^	β waves: 12.75-30 Hz^25^^,^^38^	High voltage slow waves: up to 40 μV^29^^,^^40^	Similar to wake^22^
***Electro-oculography***	High amplitude and frequency^25^^,^^38^^,^^41^	Decreased amplitude and frequency^25^^,^^38^^,^^41^	No or low amplitude activity^25^^,^^38^^,^^41^	Rapid eye movements^25^^,^^38^^,^^41^
				Increased activity^22^
***Electromyography***	High activity^41^	Reduced but observable activity^41^	Decreased nuchal activity compared to wake^22^	Low amplitude activity,^29^ except for muscle twitches^31^
				Absence of muscle activity^41^

Table modified from Niinikoski, I. Sleep-disordered breathing and inflammatory response in dogs. Dissertation. University of Helsinki. 2024. https://hdl.handle.net/10138/575935. Abbreviations: μV = microvolt; Hz = hertz; NREM = non-rapid eye movement; REM = rapid eye movement.

Sleep in dogs is polyphasic, that is, sleep cycles are not linked back-to-back to form 1 long period of sleep.^25^ Rather, sleep is divided into smaller segments throughout the day and night with time spent awake in between.^25^ A sleep cycle includes all 3 sleep stages and averages between 20 and 56 min.^25^^,^^31^^,^^45^^,^^48^ Between 2 successive wake periods, the dog sleeps on average between 47 and 127 min.^47^ This sleep segment usually contains 2 REM sleep phases.^47^

## Sleep-disordered breathing

### Classification and pathogenesis of sleep-disordered breathing

The umbrella term SDB describes all breathing problems during sleep.^49^ In humans, SDB is classified into OSA, central sleep apnea (CSA), sleep-related hypoventilation, and sleep-related hypoxemia.^49^ Naturally occurring SDB, resembling OSA in humans, was first described in English Bulldogs in 1987.^4^ Regarding the literature in dogs, the term SDB is often used rather than differentiating between obstructive and central backgrounds of the disorder. This article will also refer to the disorder in humans as OSA and in dogs as SDB. Although some centrally occurring SDB episodes are described in a few English Bulldogs,^4^^,^^50^ the vast majority of apneas and hypopneas are of obstructive origin,^4^^,^^12^^,^^14^ and obstructive SDB seems indeed to be the most common form in dogs. Sleep-related hypoventilation and sleep-related hypoxemia remain unstudied in dogs.

Many phenomena make the upper airways susceptible to collapse during sleep. During sleep, the main stimulus for ventilation is elevated arterial partial pressure of carbon dioxide, which leads to a rise in brain extracellular hydrogen ion concentration and accelerated ventilation. Ventilatory responses to hypoxemia and hypercapnia during sleep are reduced compared to wake.^40^ The upper airways lack bony structures, which increases the likelihood of obstruction, especially during muscle atonia in REM sleep.^5^

Brachycephaly, the shortened and flattened skull type produced by decades of selective breeding, is associated with obstruction in breathing during both wake and sleep.^51^^,^^52^ The altered growth of the basioccipital and basisphenoid bones results in abnormal growth of the skeletal muzzle^1^ and there is a mismatch of tissue in the available space due to the lack of a corresponding soft tissue decrease.^51^^,^^53^ Brachycephalic obstructive airway syndrome (BOAS) is common in brachycephalic dogs.^53^ In BOAS, narrow nostrils,^54–56^ oversized nasal turbinates,^57^^,^^58^ and an elongated soft palate^54^^,^^57^ obstruct airflow in the reduced space. In addition, a hypoplastic trachea^57^ and macroglossia^59^ can be associated with BOAS. Everted tonsils,^54^ everted laryngeal saccules,^54^ laryngeal collapse,^57^^,^^60^ and bronchial collapse^61^ can further impede airflow.

During sleep, upper airway dilator muscle activity is reduced, resulting in a smaller pharyngeal opening in the already narrow pharynx. Upper airway collapse leads to diminished or absent airflow, reduced arterial partial pressure of oxygen and elevated arterial partial pressure of carbon dioxide. Peripheral chemoreceptors are activated and ventilatory effort rises.^62^ The obstruction is alleviated as the upper airway muscles rapidly contract after awakening, and normal breathing resumes.^49^ The cycle continues when sleep recommences.

Fibrosis and excessive connective tissue in the sternohyoid, an upper airway dilator muscle, are reported in the English Bulldog.^63^ Conversion to a faster myosin heavy chain II phenotype in the sternohyoid of the English Bulldog suggests repeated stimulation and contraction during arousals related to SDB.^63^ Repeated arousals result in fragmentation of sleep and changes in sleep macrostructure. A higher amount of REM sleep, possibly because of REM fragmentation and the need for rebound REM sleep, is reported in brachycephalic dogs.^64^

In central SDB, a loss of output from the respiratory center responsible for generating respiratory rhythm leads to absent ventilation and loss of respiratory output.^65^ Although most SDB events in dogs are of obstructive origin,^4^^,^^12^^,^^14^ centrally occurring apneas and hypopneas have been described in a small cohort of English Bulldogs.^4^^,^^50^ However, these dogs also exhibited obstructive SDB episodes,^4^^,^^50^ and primary central SDB has not been described in dogs.

### Diagnostic methods

Polysomnography is the gold standard for the diagnosis of SDB. It includes EEG, electro-oculography, electromyography, electrocardiography, and monitoring of airflow, respiratory effort, and blood oxygenation.^66^ Polysomnography has been used in dogs, both in sleep studies^25^^,^^67^ and in assessment of SDB,^4^^,^^5^^,^^50^^,^^68–70^ but use is predominantly limited to research purposes. The method has challenges, as the reliability of pulse oximetry for the screening of hypoxemia in non-anesthetized dogs is questionable^71^ and airflow monitoring in dogs is difficult. However, successful use of both oximetry and nasal airflow measurement in polysomnography studies assessing breathing during sleep is described.^4^^,^^5^^,^^50^^,^^68–70^

Whole-body barometric plethysmography, where the dog rests in a chamber and its respiratory efforts induce pressure oscillations proportional to tidal volume, has been used for screening SDB in a small number of dogs.^12^ Panting prevented evaluation of data in 2 out of 5 dogs in this study.^12^

Both polysomnography and whole-body barometric plethysmography require extensive equipment and expertise, and for the dog to be able to sleep in a laboratory setting. The first-night effect, that is, unfamiliar surroundings causing changes in sleep macrostructure, is a known concern in laboratory sleep studies.^72^ However, only polysomnography offers exact information on oxygenation, sleep stages, and whether the dog is awake or asleep.

Recently, a portable neckband system was used for determination of SDB in dogs.^13^^,^^14^ The neckband combines a piezoelectric microphone for tracheal sounds, an ambient microphone, and a gyroscope for position and movement data.^13^^,^^14^ The device was used at the dog’s home and allowed for noninvasive assessment of SDB events.^13^^,^^14^ The device cannot be used in dogs with a neck girth less than 25 cm or greater than 65 cm and does not provide data on airflow, oxygenation, or whether the dog is asleep or awake.^13^^,^^14^

Questionnaire screening tools are used to assess subjective symptoms, such as sleepiness, and clinical signs, including snoring, of OSA in humans.^73^^,^^74^ Questionnaires on owner-perceived signs of SDB and sleeping issues have been used in dogs.^14^^,^^28^^,^^75–77^ Daytime sleepiness can be difficult for owners to identify, as sleeping during the day is normal in dogs.^25^ In addition, determining whether the dog is asleep or awake during the drowsiness stage is unclear without EEG.^25^ Owners can consider snoring normal for brachycephalic dogs^28^^,^^77^ and it can be difficult to distinguish from wake breathing patterns.

Currently, no consensus guidelines for the assessment of SDB and the scoring of SDB severity in dogs exist. In humans, an apnea is defined as a ≥ 90% decrease in airflow, lasting at least 10 s.^49^ Hypopnea definitions are more mixed, with the current recommendation being a ≥ 30% decrease in airflow associated with a ≥ 3% decrease in oxygen saturation or an arousal.^78^ In addition, respiratory effort-related arousals, breath sequences which lead to arousal but do not meet diagnostic criteria for apnea or hypopnea, are not always included in the summary of SDB.^11^ There are few reports of respiratory effort-related arousals in dogs.^50^^,^^69^ The apnea–hypopnea index summarizes the number of apnea and hypopnea events per hour of sleep.^11^^,^^49^ The apnea–hypopnea index can only be calculated from polysomnography studies, where wake can be distinguished from sleep by EEG.^11^ In non-polysomnographic studies, such as the portable neckband system, a respiratory event index is used to summarize the number of apneas and hypopneas per hour of estimated sleep.

### Signs and clinical findings

Recognizing signs related to SDB can be challenging also due to the frequent normalization of clinical signs related to brachycephaly by owners of brachycephalic dogs.^79^ Owners of brachycephalic dogs are significantly more likely to agree that sleeping sitting up is normal compared to owners of non-brachycephalic dogs.^79^ This might be partly explained by cognitive dissonance, where psychological discomfort prompts the owner to deny a problem, such as difficult breathing, in their own pet, while being aware of the issue on a larger scale within the brachycephalic breed.^77^

Signs and diagnostic findings of potential SDB brachycephalic dogs are presented in Table 2.

**Table 2:** Signs and diagnostic findings of potential sleep-disordered breathing in brachycephalic dogs.

**Signs**	**Diagnostic findings**
**Increased upper respiratory sounds and snoring^4^^,^^12–14^^,^^80^^,^^81^^,^^a^**	Hypoxemia during sleep, evaluated by pulse oximetry^4^^,^^5^^,^^50^^,^^68–70^
**Apneic episodes^12^^,^^14^ with cyanosis^82^**	Hypoxemia during wake, evaluated by arterial blood gas analysis^4^^,^^83–86^^,b^
**Sleeping with chin elevated^12^^,^^87^**	Higher hemoglobin^85^^,^^88^ especially in older BDs^85^^,b^
**Sleeping sitting up^12^^,^^14^^,^^87^**	Higher packed cell volume^85^^,b^
**Sleeping with mouth open^14^^,^^87^^,^^89^**	Hypercoagulability^88^
**Choking sounds^4^^,^^87^ and gasping while sleeping^89^**	Higher mean^84^ and systolic^90^ blood pressure
**Restless sleep,^14^ frequent arousals,^4^^,^^89^ and sleeping very little^87^**	Cardiac troponin 1 over reference range in most BDs^90^
**Hypersomnolence^4^^,^^12^^,^^82^**	Higher right heart pressures and lower systolic and diastolic ventricular function^91^

Abbreviations: BD = brachycephalic dog; SDB = sleep-disordered breathing.

a Snoring does not seem to correlate with degree of SDB in BDs.^13^

b No difference in hemoglobin, hematocrit, and saturation between wake BDs and normocephalic dogs in other studies.^83^^,^^92^

The most common owner-perceived signs of SDB in a study objectively assessing SDB with a portable neckband were snoring, restless sleep, sleeping sitting up or with toy in mouth, and apneic episodes during sleep.^14^ Owner-perceived signs of SDB are more prevalent in dogs with more than 5 SDB events per hour of estimated sleep.^14^ In this study, all owners of dogs with more than 15 SDB events per hour of estimated sleep reported apneic episodes.^14^

Snoring is not always recognized by owners of brachycephalic dogs.^14^ Objectively measured, brachycephalic dogs snore more than normocephalic dogs, but snoring can occur in the absence of SDB in both brachycephalic and normocephalic dogs.^14^ The amount of time spent snoring during the night did not correlate with the degree of SDB in brachycephalic dogs in a study utilizing the portable neckband,^13^ and additional diagnostics are needed for assessment of SDB in these breeds.

It is suggested that the changes in red blood cell variables described in brachycephalic dogs result from hypoxemia-induced erythropoietin production.^85^ Higher hemoglobin values in older brachycephalic dogs might be suggestive of more severe recurring hypoxemia with aging.^85^ However, other studies report no difference in hemoglobin, hematocrit, and hemoglobin oxygen saturation in venous blood samples between normocephalic and brachycephalic dogs.^83^^,^^92^

Although in humans, the correlation between OSA and cardiovascular and thromboembolic disease is well known,^6^^,^^93^ the association in dogs is not clear. Atrial structure remodeling occurs in an induced dog model of obstructive SDB^94^ and cardiac troponin I, an indicator of myocardial damage, was above the reference range in 47.8% of brachycephalic dogs in a study.^90^ In OSA in humans, myocardial damage is suspected to result from the elevated sympathetic activation and low-grade inflammation due to sleep fragmentation and sleep deprivation.

### Risk factors

The major risk factor for SDB is brachycephaly,^4^^,^^14^ the shortened and flattened skull type achieved through decades of selective breeding.^52^ Sleep-disordered breathing is reported in brachycephalic dogs in studies analyzing breathing during sleep both objectively^4^^,^^5^^,^^12–14^^,^^50^^,^^68–70^ and subjectively.^82^^,^^89^^,^^95^

Increasing severity of BOAS is a risk factor for SDB.^14^ Severity of BOAS is graded by assessing breathing and body temperature before and after exercise.^80^^,^^96^ Dogs are classified into grade 0 (no signs of BOAS), grade 1 (mild signs), grade 2 (moderate signs), or grade 3 (severe signs). Dogs with no or mild signs of BOAS (grades 0 and 1) are considered BOAS negative and dogs with moderate or severe signs (grades 2 and 3) BOAS positive.

Sleep-disordered breathing is objectively described also in the normocephalic Norwich Terrier.^14^ Norwich Terriers are prone to upper airway syndrome, where the constricted laryngeal region and lymphoedema, instead of brachycephaly, lead to upper airway obstruction.^97–99^

Excess weight (body condition score greater than 5/9) is a risk factor for both SDB^14^ and BOAS.^96^^,^^100^^,^^101^ Obesity decreases minute volume and limits airflow in brachycephalic dogs^100^ and worsens respiratory measurements, such as tidal volume, also in normocephalic dogs.^102^ Respiratory function and oxygenation improve after weight loss in normocephalic dogs,^103^ which may also be reflected in their quality of sleep.

Sex and aging are not identified as risk factors for SDB,^14^ although both are associated with OSA in humans. In humans, hypothyroidism is associated with OSA.^104^ Chiari malformation is associated with CSA in people,^105^ but this association has not been evaluated in dogs. Although SDB is described in breeds where Chiari-like malformation is prevalent, including Cavalier King Charles Spaniels^12^^,^^14^ and a Chihuahua,^82^ the association between SDB and Chiari-like malformation in dogs needs further investigation. In Cavalier King Charles Spaniels, the objectively measured SDB events have been of obstructive origin.^12^^,^^14^

Certain sedative medications are associated with SDB events in humans. Gabapentin, commonly used for chronic pain in dogs, increases the number of apneas and hypopneas in humans. In addition, opioids are associated with central SDB events in humans.^106^ The relationship between SDB and medications has not been evaluated in dogs.

### Treatment

Treatment of SDB can be surgical or conservative. Since obstructive SDB is closely linked to brachycephaly, surgical interventions aimed at addressing factors contributing to upper airway obstruction and BOAS are likely to improve signs of SDB. Numerous surgical procedures for alleviation of signs of BOAS are described. These include widening the nares and nasal vestibules,^107^ removal of nasal conchae,^108^ shortening and thinning the soft palate,^87^ and removal of everted laryngeal saccules and enlarged tonsils.^87^

A radical reduction in the number of SDB events, from 48 to 0 per hour, after BOAS surgery was reported in one English Bulldog in a polysomnographic study.^70^ Owner-reported incidence of apneic episodes^87^ and other owner-perceived signs of SDB^12^ decrease after multilevel BOAS surgery. However, continuation of SDB after BOAS surgery is described both objectively^13^^,^^14^ and subjectively.^87^^,^^95^ In addition, permanent tracheostomy improves quality of life in dogs with SDB.^109^

Conservative treatment forms include medications, weight loss, and potentially continuous positive airway pressure (CPAP) and positional therapy. Treatment trials are few and in a limited number of dogs.

Medical interventions, aimed at stimulating upper airway motoneurons and increasing upper airway dilator muscle activity via 5-HT receptors, have been used in individual brachycephalic dogs without owner-perceived SDB^82^^,^^89^ and small groups of dogs in clinical trials where SDB severity was assessed by polysomnography.^50^^,^^68^^,^^69^

Ondansetron, a serotonin antagonist, reduces the number of apneas and hypopneas in REM sleep in English Bulldogs.^50^ However, it does not affect time spent in oxygen desaturation or fully resolve SDB.^50^ Successful use of ondansetron is described in case reports in 2 dogs with suspected SDB.^82^^,^^89^ Ondansetron is not FDA-approved for treatment of SDB in dogs. It has been used at a dose of 0.7-1 mg/kg PO q12h.^50^^,^^82^^,^^89^

Trazodone, a mixed serotonin agonist and antagonist, combined with serotonin precursor L-tryptophan, reduces the number of SDB events in NREM sleep in English Bulldogs.^69^ However, the effect on SDB events in REM sleep, where the majority of obstructive SDB events occur, is minor.^69^ This, combined with the sedative effect of trazodone and associated hypersomnolence, likely limits its usability in treatment of SDB in dogs.

A rare but serious adverse effect of serotonergic medications is serotonin toxicosis, where excessive amounts of serotonin concentrate in the central nervous system.^110^ Dogs with a mutation in the ABCB1 gene are more likely to be at risk for toxicosis related to ondansetron.^111^ However, brachycephalic breeds are not listed as high-risk breeds for this mutation.^112^ Since BOAS is associated with severely dysfunctional thermoregulation, brachycephalic dogs could be more at risk for hyperthermia related to serotonin syndrome.

Intranasal administration of corticosteroids,^113^ nasal dilatators,^114^ and surfactant administration via nasal catheher^115^ are used for treatment of OSA in humans with varying results. No studies exist in dogs, and while intranasal administration of corticosteroids is feasible, the use of nasal dilators and surfactant administration by nasal catheter in conscious dogs is likely impractical.

A tetanus neurotoxin injection into the geniohyoid muscle of an English Bulldog significantly reduces the number of apneas and hypopneas during sleep.^116^ Injection of tetanus toxin and an antibody trap into the rostral geniohyoid muscle of 6 English Bulldogs decreases BOAS severity grade for 20-53 weeks.^117^ The degree of SDB was not evaluated in this study.^117^ Although half of the owners report improved breathing during wake, constant or occasional snoring is described by owners in all dogs also after the injection.^117^

Weight loss improves respiratory function in healthy dogs by improving lung capacity and oxidative status.^103^ Excess weight is a risk factor for SDB in both brachycephalic and normocephalic dogs.^14^ In humans, weight loss is an important treatment modality of OSA and is recommended for obese and overweight OSA patients. In addition, weight reduction decreases severity of OSA in humans.^118^^,^^119^

The gold standard for the treatment of OSA in humans is CPAP. The CPAP device provides continuous positive airway pressure during inspiration and expiration and thus prevents upper airway collapse.^120^ Continuous positive airway pressure reduces the amount of SDB events markedly, with complete resolution of apneas and hypopneas in some patients.^121^ In humans, subjective evaluations of quality of life and sleepiness improve with CPAP treatment.^122^ In dogs, CPAP provided by a pediatric helmet has been used in sedated healthy dogs,^123^ normocephalic^124^ and brachycephalic^125^ dogs recovering from general anesthesia, and in severely ill dogs for treatment of acute cardiogenic pulmonary edema^126^ and hypoxemic acute respiratory failure.^127^ The feasibility of CPAP for treatment of SDB is questionable, as familiarization to the device would require great effort.^95^ However, CPAP was used successfully for years in a Cavalier King Charles Spaniel with severe owner-perceived signs of SDB.^95^ Although the facial conformation of brachycephalic dogs might not allow for a sufficient fit of the mask, which is designed for humans, in this individual, the induction mask was secured with a soft muzzle.^95^ A pediatric helmet could be convenient in brachycephalic breeds, but thermoregulatory risks might be considerable. It should be noted that in humans adherence to CPAP use varies,^128^ and positive response to treatment requires consistent nightly use.^129^

Gravity can exacerbate upper airway collapsibility in certain positions. Elevation of the head reduced owner-perceived signs of SDB in 2 dogs.^82^^,^^89^ In humans positional therapy, that is, preventing sleeping in a supine position, is effective in some OSA patients.^130^

## Sleep-disordered breathing and low-grade inflammation

Inflammation is a key part of immunity. However, chronic low-grade inflammation increases total mortality rate^131^ and is an independent risk factor for multiple chronic disorders, such as neoplasia^9^ and coronary heart disease^10^ in humans. Chronic low-grade inflammation is associated with numerous disease processes, including OSA^132^ and diabetes mellitus,^133^ in humans.

### Hypoxemia-induced inflammation

In OSA, low-grade inflammation is assumed to result from intermittent hypoxia—a lack of oxygen at the tissue level—due to hypoxemia, which is an abnormally low blood oxygen concentration. This is caused by the repeated apneic and hypopneic episodes during sleep.^134^ The hypoxia signaling pathway, primarily governed by the hypoxia-inducible factor, is activated by the decreased oxygen concentration. The activated hypoxia-inducible factor complex regulates many immune cell functions, such as cell motility,^135^ migration,^136^ and proliferation.^137^ Hypoxia-inducible factor is also involved in upregulation of various cytokines^138^ and chemokines.^139^ In addition to disease processes, hypoxemia-induced low-grade inflammation also occurs in healthy dogs due to high altitude.^140^

The relationship between chronic low-grade inflammation and OSA is widely researched in humans, but inflammatory mediators have not been assessed in dogs with relation to objectively measured SDB. Although in humans, OSA is associated with comorbidities, including obesity and cardiovascular disease, which also induce low-grade inflammation, the severity of OSA correlates with elevated concentration of various inflammatory mediators.^141^^,^^142^

Current knowledge of low-grade inflammation in brachycephalic dogs, presumably as a result of intermittent hypoxemia resulting from upper airway obstruction, is compiled in Table 3. These changes are not evaluated with respect to degree of SDB.

**Table 3:** Summary of literature assessing inflammation in brachycephalic dogs.

**Measure of inflammation**	**Type of inflammatory mediator**	**In BDs compared to normocephalic control dogs or other specified group**	**In BDs with different severity of BOAS signs**	**Methodology**
***CCL2***	Pro	NC in both serum and bronchoalveolar lavage fluid^17^	NC in serum^17^	Solid phase immunoassay^17^
***CRP***	Pro	NC in plasma^92^ NC compared to reference values, in serum^143^ ↑ than reference range in 51.9% of BDs, in serum^16^	NC in serum^90^ NC before and after BOAS surgery, in serum^143^	Immunoturbidimetric assay^90^^,^^92^^,^^143^
***Glutathione peroxidase***	Anti		NC between BDs with different levels of upper airway obstruction before or after BOAS surgery, in heparinized whole blood^144^	Spectrophotometry^144^
***Haptoglobin***	Pro	NC compared to reference range, in serum^90^	NC in serum^90^ ↑ after BOAS surgery, in serum^143^^,^^a^	Colorimetry^90^^,^^143^
***Eosinophil***	Pro	↑^145^ ↓ in K_2_-EDTA^92^		
***IL-1β***	Pro	NC in plasma^15^		ELISA^15^
***IL-6***	Pro	NC in plasma^15^	↓ with moderate signs of BOAS compared to controls, in plasma^15^	ELISA^15^
***IL-10***	Anti	↑ in plasma^15^		ELISA^15^
***IL-13***	Anti	↑ in plasma^15^	↑ with no signs of BOAS compared to controls, in plasma^15^	ELISA^15^
***IL-17A***	Pro	NC in plasma^15^	↑ with severe signs of BOAS and requiring surgery compared to controls and BDs with no or moderate signs of BOAS, in plasma^15^	ELISA^15^
***Lymphocyte***	Pro	↓ in K_2_-EDTA^92^		
***Malondialdehyde***	Pro		NC between BDs with different levels of upper airway obstruction before or after BOAS surgery, in plasma^144^	Liquid chromatography^144^
***Monocyte***	Pro	↑^145^ NC in K_2_-EDTA^92^		
***Neutrophil***	Pro	↑^145^ NC in K_2_-EDTA^92^	Positive correlation with BOAS severity^145^	
***Neutrophil to leucocyte ratio***	Pro	↑^145^ ↑ in K_2_-EDTA^92^	Positive correlation with BOAS severity^145^	
***Nitric oxide***	Anti-inflammatory under normal physiological conditions; proinflammatory in abnormal conditions^146^	↑ in plasma^15^ ↑ in plasma^92^	↑ with severe signs of BOAS and BDs requiring treatment compared to controls, in plasma^15^	Griess reaction (colorimetry)^15^ Chemiluminecence^92^
***Superoxide dismutase***	Anti		↑ in BDs with no or very mild upper airway obstruction compared to BDs with moderate or severe obstruction, in heparinized whole blood^144^ ↑ in BDs with moderate or severe upper airway obstruction after BOAS surgery compared to before, in heparinized whole blood^144^	Spectrophotometry^144^
***TNF-α***	Pro	↑ in plasma^15^		ELISA^15^
***VEGF-A***	Pro	↑ in English Bulldogs but not French Bulldogs or Pugs compared to controls, in serum^17^ ↓ in English Bulldogs compared to controls, in bronchoalveolar lavage fluid^17^	NC in serum^17^	Solid phase immunoassay^17^

Table modified from Niinikoski, I. Sleep-disordered breathing and inflammatory response in dogs. Dissertation. University of Helsinki. 2024. https://hdl.handle.net/10138/575935. Abbreviations: ↑ = increase; ↓ = decrease; BD = brachycephalic dog; BOAS = brachycephalic obstructive airway syndrome; CCL2 = C-C motif chemokine ligand 2; CRP = C-reactive protein; EDTA = ethylenediaminetetraacetic acid; ELISA = enzyme-linked immunosorbent assay; IL = interleukin; NC: no change; TNF = tumor necrosis factor; VEGF-A = vascular endothelial growth factor A.

a Likely due to postoperatively given corticosteroid medication.

## Conclusions and future directions

Adequate, good-quality sleep is an important component of welfare. Several actions can be taken in order to diminish SDB, which occurs primarily in brachycephalic but also in normocephalic dogs. Although more research into the comorbidities and consequences of SDB is needed, the most important risk factor for SDB is brachycephaly.^4^^,^^5^^,^^12–14^^,^^50^^,^^68–70^ In addition to brachycephaly, moderate or severe signs of BOAS and excess weight predispose to SDB.^14^ The relationship between SDB and low-grade inflammation needs further research.

SDB is, at least partly, treatable with conservative and surgical interventions. The identification of dogs in need of treatment is crucial. In terms of methods for simple diagnostics, owner-perceived signs, such as sleeping sitting up or with a toy in mouth, nighttime restlessness, and apneic episodes during sleep can indicate SDB. However, further diagnostics are needed to objectively diagnose SDB. Snoring cannot be used as the sole indicator of SDB, as it occurs even in the absence of apneas and hypopneas and does not correlate with the rate of SDB events in brachycephalic dogs.^13^^,^^14^ The currently available objective diagnostic methods are not easily adapted into clinical practice. Although the use of a portable neckband system for diagnostics of SDB is described,^13^^,^^14^ it is not commercially available at present. More research on easily used diagnostic methods, diagnostic criteria, and cut-off values between a normal and an abnormal number of SDB events during sleep are needed to enhance our understanding of sleep and SDB in dogs. Practical diagnostic methods would also allow for assessment of treatment response. Currently, neither surgical nor conservative methods completely resolve SDB in all dogs. Nonetheless, maintaining optimal weight is advisable in all dogs to enhance respiratory function.

The consequences of brachycephaly on the welfare of dogs are severe. The effect of crossbreeding brachycephalic dogs with normocephalic dogs on breathing during sleep warrants further investigation. The future of brachycephalic breeds needs to be critically evaluated.

## Off-label antimicrobial declaration

The authors declare no off-label use of antimicrobials.

## Institutional animal care and use committee or other approval declaration

The authors declare no institutional animal care and use committee or other approval was required.

## Abbreviations

BOAS: brachycephalic obstructive airway syndrome
CSA: central sleep apnea
CPAP: continuous positive airway pressure
EEG: electroencephalography
NREM: non-rapid eye movement
OSA: obstructive sleep apnea
REM: rapid eye movement
SDB: sleep-disordered breathing
